# Improved Protocol for Rapid Identification of Certain *Spa* Types Using High Resolution Melting Curve Analysis

**DOI:** 10.1371/journal.pone.0116713

**Published:** 2015-03-13

**Authors:** Benjamin Mayerhofer, Anna Stöger, Ariane T. Pietzka, Haizpea Lasa Fernandez, Bernhard Prewein, Sieglinde Sorschag, Renate Kunert, Franz Allerberger, Werner Ruppitsch

**Affiliations:** 1 University of Applied Sciences Wiener Neustadt, Department of Biomedical Analytics, Wiener Neustadt, Austria; 2 Austrian Agency for Health and Food Safety, Institute of Medical Microbiology and Hygiene, Vienna, Austria; 3 KABEG, Klinikum Klagenfurt, Institute of Laboratory Diagnostics and Microbiology, Klagenfurt, Austria; 4 University of Natural Resources and Life Sciences, Department of Biotechnology, Institute of Applied Microbiology, Vienna, Austria; Rockefeller University, UNITED STATES

## Abstract

Methicillin-resistant *Staphylococcus aureus* is one of the most significant pathogens associated with health care. For efficient surveillance, control and outbreak investigation, *S*. *aureus* typing is essential. A high resolution melting curve analysis was developed and evaluated for rapid identification of the most frequent *spa* types found in an Austrian hospital consortium covering 2,435 beds. Among 557 methicillin-resistant *Staphylococcus aureus* isolates 38 different *spa* types were identified by sequence analysis of the hypervariable region X of the protein A gene (*spa*). Identification of *spa* types through their characteristic high resolution melting curve profiles was considerably improved by double spiking with genomic DNA from *spa* type t030 and *spa* type t003 and allowed unambiguous and fast identification of the ten most frequent *spa* types t001 (58%), t003 (12%), t190 (9%), t041 (5%), t022 (2%), t032 (2%), t008 (2%), t002 (1%), t5712 (1%) and t2203 (1%), representing 93% of all isolates within this hospital consortium. The performance of the assay was evaluated by testing samples with unknown *spa* types from the daily routine and by testing three different high resolution melting curve analysis real-time PCR instruments. The ten most frequent *spa* types were identified from all samples and on all instruments with 100% specificity and 100% sensitivity. Compared to classical *spa* typing by sequence analysis, this gene scanning assay is faster, cheaper and can be performed in a single closed tube assay format. Therefore it is an optimal screening tool to detect the most frequent endemic *spa* types and to exclude non-endemic *spa* types within a hospital.

## Introduction

Methicillin-resistant *Staphylococcus aureus* emerged in the early 1960s and became a leading cause of healthcare associated infections (HA-MRSA) worldwide [1]. The situation worsened with the emergence of highly pathogenic MRSA clones in the community (CA-MRSA for community acquired MRSA) [2] and in livestock (LA-MRSA for livestock associated MRSA) [3, 4]. Molecular characterization of MRSA clones is an indispensable prerequisite for outbreak investigation and epidemiological studies. The most widely used techniques for MRSA typing are pulsed-field gel electrophoresis (PFGE), multi-locus sequence typing (MLST), and sequence based typing of the variable X-region of the staphylococcal protein A gene (*spa* typing) [5–7]. With respect to simplicity, speed, accuracy, data interpretation, discrimination, usability for short term outbreak investigation and long-term epidemiological studies, and in view of automation of analysis facilitated through the Ridom Staphserver software [8] *spa* typing is currently the most widely used method for MRSA strain typing [7, 8]. The Ridom *spa* server database currently contains 14,334 *spa* types with data from 112 countries worldwide (http://www.spaserver.ridom.de, 2014-11-25). Nevertheless, sequence based typing approaches are still expensive and laborious. PCR based typing approaches are more suitable for the clinical laboratory in terms of speed and cost [9]. High resolution melting (HRM) curve analysis represents a powerful method for single nucleotide polymorphism based strain differentiation [10, 11]. The principle of HRM is that sequence variations within a DNA fragment alter the melting behavior of the double stranded DNA, finally resulting in a characteristic and specific melting curve profile. Single nucleotide changes within DNA can be divided into four single nucleotide polymorphism (SNP) classes, characterized by different melting temperature (Tm) shifts [12, 13]. SNP class 1 is characterized by C/T and G/A, SNP class 2 by C/A and G/T, SNP class 3 by C/G, and SNP class 4 by A/T base exchanges. SNP class 1 and 2 mutations can easily be detected by HRM due to melting temperature differences of more than 0.5°C, whereas detecting SNP class 3 and 4 mutations may be more challenging due to smaller melting temperature differences (<0.4°C for SNP class 3 and <0.2°C for SNP class 4) [13]. The addition of defined DNA material to the reaction mix (spiking) can improve the separation of closely related or even similar melting curves through the formation of heteroduplex DNA, which display more distinct melting properties compared to homoduplex DNA strands [14].

In this study we evaluated high resolution melting curve analysis (HRM-CA) of *spa* [15, 16] as a rapid, accurate and cost effective typing tool of MRSA isolates obtained from a hospital consortium and we were able to improve this method using a double spiking approach.

## Material and Methods

### Microorganisms

Five hundred and fifty-seven methicillin resistant *Staphylococcus aureus* isolates were sent for *spa* typing to the national staphylococcal reference laboratory at the Austrian Agency for Health and Food Safety from 2006 to 2010 from a hospital consortium consisting of six hospitals in an Austrian province. The consortium covered a total of 2,435 beds (systemized number of beds for general class; median 224/291 beds, range: 100–1,447 beds). Thirty-eight *spa* types could be identified by *spa* sequence analysis. The ten most frequent *spa* types representing 93% of all isolates were t001 (58%), t003 (12%), t190 (9%), t041 (5%), t022 (2%), t032 (2%), t008 (2%), t002 (1%), t5712 (1%) and t2203 (1%). The remaining *spa* types, in order of decreasing frequency, were t011, t1607, t012, t020, t045, t068, t116, t2689, t2954, t513, t009, t015, t026, t030, t037, t094, t127, t1301, t149, t1491, t1607, t3220, t4342, t651, t655, t892, t919, t7549.

In this study 212 arbitrarily chosen MRSA isolates comprising all 38 *spa* types were typed by high resolution melting curve analysis. Out of the 212 tested MRSA isolates 20 MRSA isolates comprising the ten most frequent *spa* types from the hospital consortium (t001 (n = 2), t003 (n = 2), t190 (n = 2), t041 (n = 2), t022 (n = 2), t032 (n = 2), t008 (n = 2), t002 (n = 2), t5712 (n = 2) and t2203 (n = 2) were used in a first step for assay development.

Genomic bacterial DNA (gDNA) was extracted from bacterial cells grown overnight at 37°C on Columbia blood agar plates (BioMérieux, Marcy I’Etoile, France) using the Quick Extract bacterial DNA extraction kit according to the manufacturer's instructions (Epicentre Biotechnologies, Madison, WI).

The concentration and quality of the purified gDNA was determined by UV spectrophotometry at 260 and 280 nm and agarose gel (1.5% wt/vol) electrophoresis.

### High resolution melting (HRM) curve analysis

The conventional PCR *spa* assay using oligonucleotides 1113F (5’- TAAAGACGATCCTTCGGTGAGC) and 1514R (5’- CAGCAGTAGTGCCGTTTGCTT) (www.ridom.de/doc/Ridom_spa_sequencing.pdf) was modified and optimized for the LightCycler LC480 instrument (Roche Diagnostics, Penzberg, Germany). All primers had an M13 sequence added to the 5′- end of the gene specific priming sequence for subsequent sequencing. PCR and HRM were performed in a single run. In a final volume of 10 μl the HRM PCR reaction mix contained 5 ng of sample gDNA, 0.35 pmol of each primer, 3 mM MgCl_2_ and the LightCycler 480 High Resolution Melting Master using ResoLight high-resolution melting dye (Roche Diagnostics, Vienna, Austria). PCR conditions were as follows: 10 min activation at 95°C, 45 cycles 95°C for 10 s, 60°C for 10 s and 72°C for 10 s and a final step at 95°C for 1 min and cooling to 40°C for 1 min. HRM was performed from 60°C to 95°C, rising at 0.02°C/s with 25 acquisitions per degree. All reactions were performed in quadruplicate using an epMotion work station (Eppendorf, Hamburg, Germany) for automatic sample preparation in 384-well microtiter plates (LC plate, Roche Diagnostics, Vienna, Austria).

Extra DNA was added to the reaction mixture (spiking) to separate spa types displaying similar or even identical melting curve profiles to improve the overall performance of the assay. Spiking results in the formation of heteroduplex amplicons that have melting curve profiles clearly distinguishable from those of homozygous amplicons. Optimal performance was achieved by adding 0.5 ng gDNA of *spa* type t003 and 0.5 ng gDNA of *spa* type t030 to the reaction mixtures.

We used the LC 480 gene scanning software version 1.5 with manual settings for sensitivity at 0.30, for temperature shift at threshold one for HRM curve analysis. After normalizing and temperature shifting the melting curves, difference plots were generated by selecting one HRM curve profile as the baseline. Only amplification products reaching the plateau phase were analyzed. For optimal performance (i.e. correct assignment of melting curve profiles to a known sequence) of HRM experiments, each run had to contain well-characterized standards (i.e. strains with known sequences) because it is not reliable to compare melting curve profiles obtained from different runs.

Using at least three different isolates of the ten most frequent *spa* types the performance of the assay was evaluated on the ViiA 7 instrument (Life Technologies, Carlsbad, CA) in combination with the MeltDoctor HRM Master Mix including Syto9 as the fluorescent dye (Life Technologies, Carlsbad, CA), and on the Rotor-Gene Q (Qiagen, Hilden, Germany) in combination with the Type-It HRM Master Mix including EvaGreen as the fluorescent dye (Qiagen, Hilden, Germany). On both instruments the settings for HRM were used as recommended by the manufacturers. Briefly, on the ViiA 7 instrument PCR conditions were a 10 min activation step at 95°C, 40 cycles 95°C for 10 s and 60°C for 1min. HRM was performed from 60°C to 95°C with a ramp rate of 0.025°C/s. On the Rotor-Gene Q PCR conditions were 10 min activation at 94°C, 42 cycles 94°C for 7 s, 64°C for 30 s and a final step at 95°C for 1 min and cooling to 40°C for 1 min. HRM was performed from 61°C to 87°C with a ramp rate of 0.02°C/s and 100 reads/s.

### Sequence analysis

The variable X region of *spa* was sequenced for all 212 MRSA isolates for HRM data verification. Prior to sequencing, amplification products were purified with EXO SAP-IT (GE Healthcare, Buckinghamshire, UK). Purified amplification product (2μl) was used for subsequent sequencing with primers M13 universal (5′-TGTAAAACGACGGCCAGT-3′) and M13 reverse (5′-CAGGAAACAGCTATGACC-3′) (Eurofins MWG Operon, Ebersberg, Germany) using the BigDye Terminator v3.1 sequencing kit (Applied Biosystems, Carlsbad, CA). DNA sequencing was performed as previously described [17]. Products were analyzed on an ABI Genetic Analyzer 3500Dx (Applied Biosystems, Carlsbad, CA) according to the manufacturer's instructions. The *spa* types were determined using Ridom StaphType software [8]. Reproducibility was tested by three persons performing the assay with the same ten most frequent *spa* types in quadruplicate. The performance of the assay was assessed by blinding the person who performed the test. The person had to identify the *spa* type by HRM analysis of *spa* from 27 uncharacterized isolates over a period of three months.

## Results

**Fig 1:**
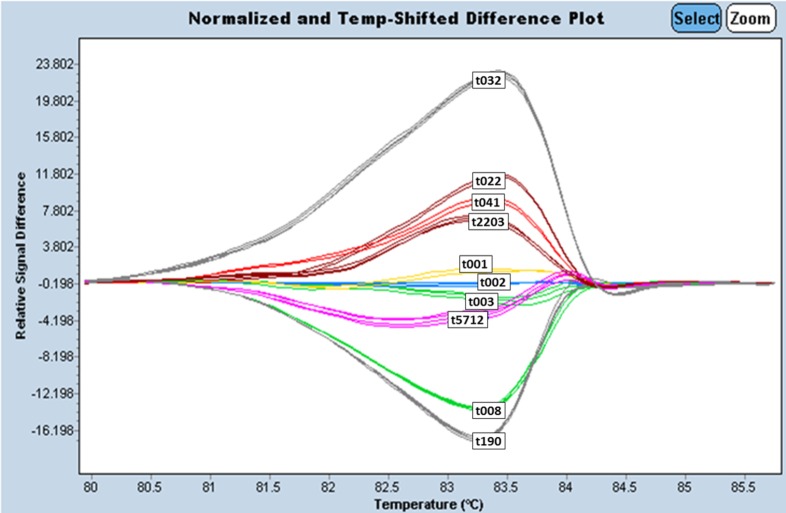
Unspiked HRM curve profiles of the ten most frequent *spa* types. t032, t022, t041, t2203, t001, t002, t003, t5712, t008, t190 on an LC 480 real-time instrument.

An HRM assay for *spa* scanning has been developed using 20 MRSA isolates of the ten most frequent *spa* types (t001 (n = 2), t003 (n = 2), t190 (n = 2), t041 (n = 2), t022 (n = 2), t032 (n = 2), t008 (n = 2), t002 (n = 2), t5712 (n = 2) and t2203 (n = 2) (with t032, t003, t002 and t008 the four most frequent (32.6%) international types included) out of an arbitrary collection of 212 MRSA isolates (Fig. 1). HRM curve analysis yielded distinct and characteristic melting curve profiles for each tested *spa* type on an LC 480 instrument, but *spa* types t001, t002 and t003 displayed closely related melting curve profiles (Fig. 1).

**Fig 2:**
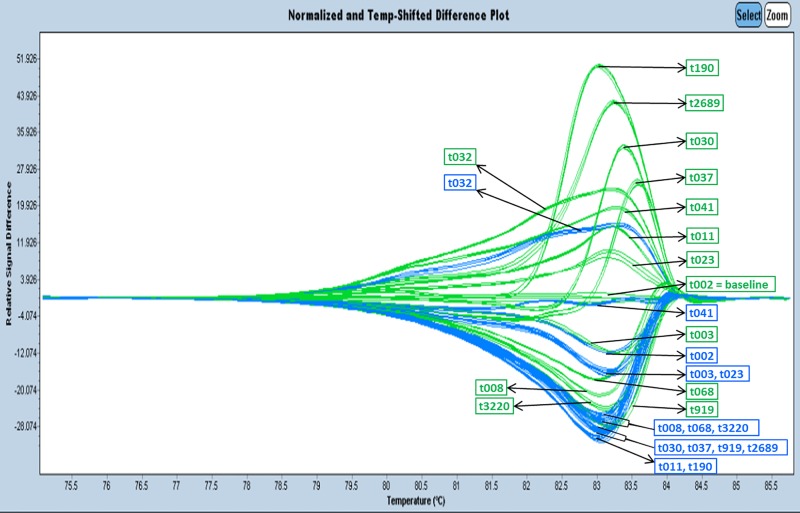
Unspiked vs. spiked HRM curve profiles. Base line spa type t002. HRM melting curve profiles in blue show unspiked samples and melting curve profiles in green show double spiked samples derived from spa types t002, t003, t008, t011, t023, t030, t032, t037, t041, t068, 190, t919, t2689, and t3220 on an LC 480 real-time instrument.

Analysis of the arbitrary collection of 192 isolates comprising all 38 endemic *spa* types revealed that *spa* type t008 shared identical melting curve profiles with t068 and t3220 (Fig. 2); t190 shared melting curve profiles with *spa* types t919, t030, t037, and the melting curve profile of t2689 was indistinguishable from the spa type t011 curve profile (Fig. 2).

**Fig 3:**
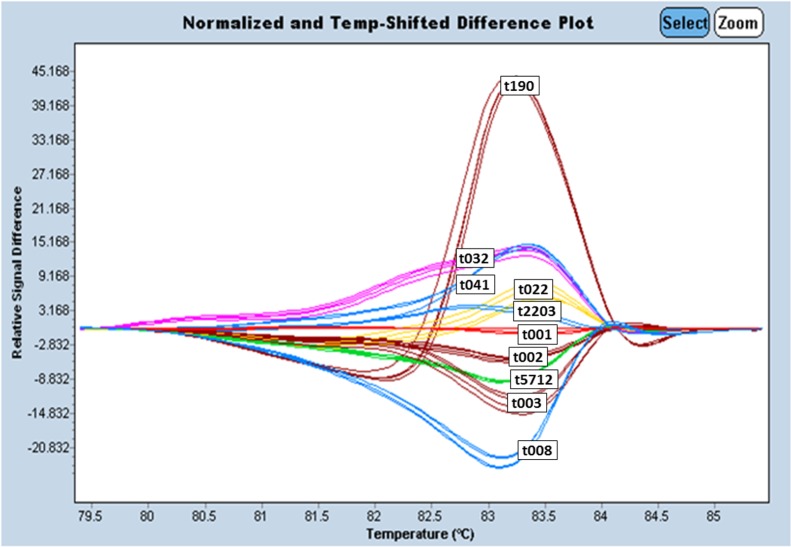
HRM curve profiles of the ten most frequent *spa* types performed on an LC 480 real-time instrument. t032, t022, t041, t2203, t001, t002, t003, t5712, t008, t190 double spiked with genomic DNA of spa types t030 and t003.

To improve the performance of the assay and to achieve a separation of *spa* types with closely related or indistinguishable melting curve profiles, reaction mixtures were individually spiked with an equal amount of genomic DNA from six arbitrarily chosen *spa* types (t001, t003, t008, t030, t190 and t3220). Single spiking with *spa* type t003 or *spa* type t030 yielded the best results but did not allow separation of all spa types (data not shown). Spiking with DNA from *spa* type t003 failed to resolve *spa* type t008 and *spa* type t068 curve profiles and spiking with DNA from *spa* type t030 failed to resolve *spa* type t001, t002 and t003 curve profiles more clearly (data not shown). Adding equal amounts of genomic DNA of *spa* types t003 and t030 to the reaction mixture resulted in optimal assay performance that allowed clear differentiation of all *spa* types, that had displayed indistinguishable melting curve profiles in preliminary experiments [t003 and t023, t041 and t2945 (data not shown), t068, t3220, t008, t030, t037, t919, t190 and t011] (Fig. 2). For a better comparison and visualisation of the spiking effect on melting curve profiles spiked and unspiked samples were analyzed on the same plate (Fig. 2). Spiking resulted in better discrimination of the ten most frequent *spa* types and of t001, t002 and t003 (Fig. 3).

The reproducibility of the assay was tested by three different persons on the subset of the 10 most frequent *spa* types. All operators were able to identify the different *spa* types and obtained similar results as shown in Fig. 3.

The performance of the assay was further evaluated by blinding the operator of the assay. Twenty-seven MRSA samples collected from the routine diagnostic laboratories from the hospital consortium were analyzed over a three-month period; the operator had no prior knowledge of the *spa* sequence analysis. A 100% correct identification rate was found for 25 samples harboring one of the ten most frequent *spa* types. Two samples not belonging to these ten most frequent *spa* types correctly displayed other specific curve profiles.

**Fig 4:**
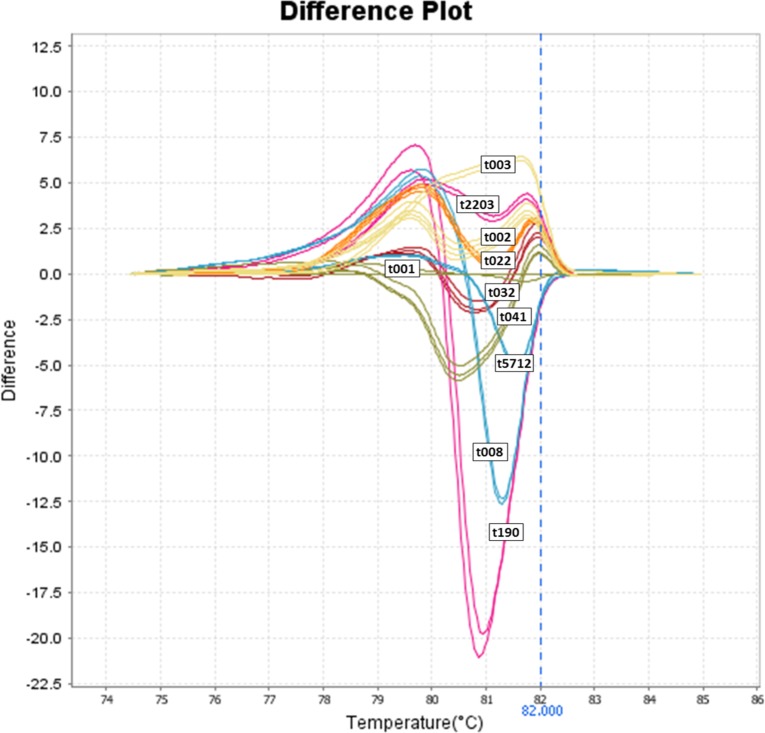
HRM curve profiles of the ten most frequent *spa* types performed on a ViiA 7 real-time instrument. t032, t022, t041, t2203, t001, t002, t003, t5712, t008, t190 double spiked with t030 and t003 genomic DNA.

**Fig 5:**
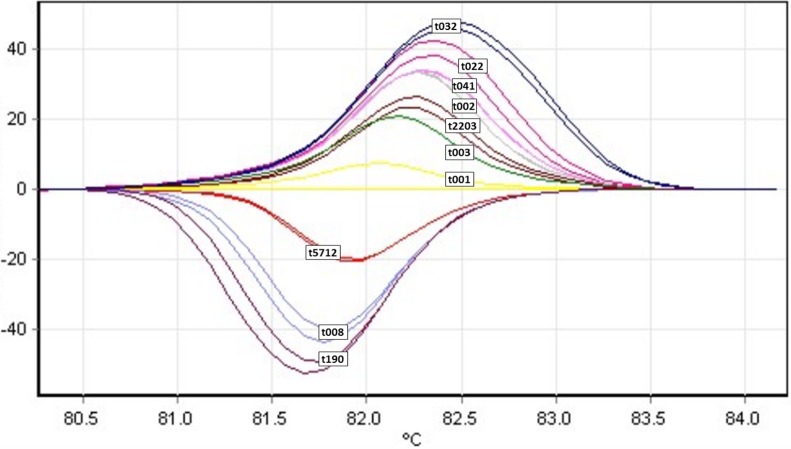
HRM curve profiles of the ten most frequent *spa* types performed on a Rotorgene Q real-time instrument. t032, t022, t041, t2203, t001, t002, t003, t5712, t008, t190 double spiked with t030 and t003 genomic DNA.

Characteristic profiles were obtained for the ten most frequent *spa* types when testing the initial 20 samples also on the ViiA 7 real-time PCR instrument and on the Rotor-Gene Q distinct melting curve (Fig. 4 and Fig. 5).

## Discussion

MRSA infections still pose a significant public health threat. Molecular analysis of MRSA is an important tool for surveillance and outbreak investigation. MLST and *spa* typing data have revealed that the MRSA population is highly clonal, showing a geographical clustering of distinct types and a limited number of MRSA clones responsible for epidemics [18, 19]. *Spa* is an outstanding genetic marker combining rapid and slow accumulating genetic variations (micro- and macrovariation), which allows long-term epidemiological studies as well as short-term outbreak investigation by analyzing a single locus [6, 7, 20, 21]. The usefulness of *spa* typing was boosted by automation of data analysis via the internet [8]. Nevertheless, although sequence based *spa* typing is extremely effective, PCR based techniques have advantages, like a single and closed tube assay format reducing the risk of contamination, the speed of real-time detection, cost effectiveness, suitability for high-throughput screening, and simplicity [15]. With the recent development of high resolution melting PCR, a useful tool has been made available for fast and accurate mutation detection [10, 11].

In addition, HRM curve profiles are highly reproducible and portable which may allow the creation of a *spa* HRM curve library [15]. However, there is to our knowledge no software available to allow direct and accurate assignment of a curve profile to a DNA sequence by comparing experimentally derived curve profiles to curve profiles stored in a database. Controls must be analyzed in parallel for accurate assignment of a curve profile to a DNA sequence or sequence type. Furthermore the sheer number of different *spa* types, currently 14,150 *spa* types registered in the SpaServer database (http://www.spaserver.ridom.de, 2014-09-25), shows that HRM curve profiling cannot replace sequence based *spa* typing. The advantage of applying HRM curve analysis is fast and accurate screening of certain DNA fragments; in this situation we have a manageable number of *spa* types. Under these premises HRM is a superior screening method for detecting endemic strains and will be useful for the surveillance of non-endemic strains and new emerging clones via the detection of new curve profiles.

Due to the detection of characteristic heterozygous curve profiles and capabilities for high-throughput colony screening HRM is a perfect tool for surveillance of mixed culture infections [22].

In this study, HRM curve analysis was able to identify correctly 38 endemic *spa* types prevalent in an Austrian hospital consortium. In contrast to recent publications [15, 16], in our study some closely related *spa* types displayed indistinguishable or highly similar curve profiles indicating that this technique has to be optimized for individual situations. The detection of single deletions or insertions by HRM curve analysis is challenging [10]. A broadly accepted solution for discriminating indistinguishable HRM curve profiles derived from different DNA fragments is the addition of extra DNA to the reaction mixture [14, 23]. This modification leads to the formation of heteroduplex amplicons that yield melting curve profiles different from those of homozygous amplicons [23]. To the best of our knowledge, we describe here for the first time a double-spiking experiment that significantly improves the performance of *spa* typing by high resolution melting. Double-spiking with genomic DNA from *spa* type t030 and *spa* type t003 yields curve profiles that allow accurate identification of all 38 *spa* types prevalent in an Austrian hospital consortium. Double spiking even yielded characteristic, unique curve profiles for *spa* types, that in the standard HRM assay had clustered in three indistinguishable groups(t008, t068 and t3220; t190, t919, t030, t037, t2689 and t011; t003 and t023; and t041 and t2945). Double spiking also improved the uniqueness of curve profiles of related *spa* types t001 (repeat profile 26-30-17-34-17-20-17-12-17-16), t002 (repeat profile 26-23-17-34-17-20-17-12-17-16) and t003 (repeat profile 26-17-20-17-12-17-17-16), facilitating an accurate identification of these *spa* types.

This assay included and allowed the differentiation of the global dominant *spa* types t032, t003, t002, t008, t011, t127, t012, t037 (in descending order according to their frequency) representing 41.1% of all isolates in Ridom’s spa server database (http://spa.ridom.de/frequencies.shtml, 2014-11-25)

The performance of the assay was also evaluated on three HRM real-time PCR instruments. On all instruments the ten most frequent *spa* types could be identified accurately. Using the LightCycler LC480 instrument as our standard platform, we found that optimal performance of each system was achieved with the HRM PCR Kit of the respective company. The melting curve profiles of the different *spa* types on the Rotor Gene Q display curve shapes similar to each other that are more difficult to distinguish—especially under screening conditions—than the difference plots from the two other instruments. However, due to limited availability of this instrument for testing, we assume that perfect HRM results can be achieved on any HRM instrument with the optimization of some parameters.

In conclusion, the optimization of a *spa* HRM assay by double spiking allows accurate, fast and cost effective differentiation of all 38 spa types and identification of the ten most frequent *spa* types derived from a hospital consortium. Compared to *spa* typing by sequence analysis (calculated for bidirectional sequencing) this HRM assay (calculated for quadruplicate sample testing) is approximately four times faster (2 vs. 8 hours) and 18 times cheaper (1.5 vs. 27 €). Due to the flexibility of the technique, adaptation to variable requirements i.e. inclusion of additional *spa* types or exclusion of *spa* types is easily possible, as is screening for or excluding certain types; manual or robotic pipetting depending on number of samples. This technique could be used for screening and rapid identification of the most frequent *spa* types of the recently emerged live-stock associated MRSA clones (ST-398, *spa* type t011 in this study) [24]. We consider the assay described here to be a valuable, efficient and rapid typing tool for MRSA surveillance and control in hospital settings that lack sequencing machines or prior to DNA sequencing.
